# Does sacubitril/valsartan work in children with heart failure?—a pilot study

**DOI:** 10.3389/fcvm.2023.1274990

**Published:** 2023-11-29

**Authors:** Yahe Xu, Mingjie Zhang, Yijun Chen, Xi Chen, Wenting Song, Limin Zhu, Liping Liu, Xiaolei Gong, Yuqi Zhang, Zhuoming Xu

**Affiliations:** ^1^ Department of Pediatric Thoracic and Cardiovascular Surgery, Shanghai Children’s Medical Center, Shanghai Jiaotong University School of Medicine, Shanghai, China; ^2^ Department of Pediatric Cardiology, Shanghai Children’s Medical Center, Shanghai Jiaotong University School of Medicine, Shanghai, China

**Keywords:** sacubitril/valsartan, heart failure, congenital heart disease, cardiomyopathy, HFrEF—heart failure with reduced ejection fraction

## Abstract

**Background:**

Sacubitril/valsartan is an angiotensin receptor neprilysin antagonist (ARNI) approved for adult heart failure (HF). Its safety and efficacy in pediatric HF patients with cardiomyopathy or congenital heart disease are poorly understood. A pilot study was conducted to assess the clinical response, efficacy and safety of sacubitril/valsartan in this population at a tertiary care hospital in China.

**Methods:**

Clinical parameters of patients who received sacubitril/valsartan from January 2019 to March 2023 were retrospectively collected and analyzed. Children over 1 month with a left ventricular ejection fraction (LVEF) <45% were included. Clinical efficacy was evaluated by echocardiographic LVEF, N-terminal pro-brain natriuretic peptide (NT-proBNP), New York Heart Association (NYHA) HF classification, HF re-admission, and death or transplantation. The initial dose was either 0.2 mg/kg bid or 0.4 mg/kg bid, with a target dose of 2.3 mg/kg bid or 3.1 mg/kg bid.

**Results:**

Forty-five patients (60% male) with a median age of 7.86 years were enrolled. Among them, 23 had congenital heart disease and 22 had cardiomyopathies. The median maintenance dose was 0.76 mg/kg. The primary endpoint of LVEF up to 45% was reached by 24 patients (53.3%). The median NT-proBNP was significantly decreased from 5,501.5 pg/ml to 2,241.5 pg/ml (*P* < 0.001), more in congenital heart disease than in cardiomyopathies (*P* = 0.032). The NYHA HF class was improved or remained stable in 42 cases (93.3%). During a median follow-up of 1.23 years, 13 patients (28.9%) were re-hospitalized due to HF, and 9 patients (20%) died or underwent transplantation. Hypotension was the main adverse event, occurring in 8 patients.

**Conclusions:**

Sacubitril/valsartan may be effective in children with HF, but its safety and outcomes may differ depending on the etiology and anatomy of HF. Early post-operative congenital heart disease patients had less tolerance, more hypotension but better recovery and outcomes, while mid- and late- post-operative congenital heart disease patients and cardiomyopathy patients had less side effects but poorer clinical outcomes.

## Introduction

Heart failure (HF) is a complex clinical syndrome resulting from diverse etiologies, with substantial clinical mortality, morbidity and treating expenses. According to the International Society for Heart and Lung Transplantation, HF in children is a clinical and pathophysiologic syndrome that results from ventricular dysfunction, volume, or pressure overload, alone or in combination. It leads to characteristic signs and symptoms, such as poor growth, feeding difficulties, respiratory distress, exercise intolerance, and fatigue, and is associated with circulatory, neurohormonal, and molecular abnormalities (1). HF in children has various etiologies, and is most commonly attributable to coexistent congenital heart disease (CHD) and cardiomyopathy (CM) (2). Current management and treatment for HF in children are generally extrapolated from therapies and guidelines in adults. The current clinical management of pediatric HF includes the use of angiotensin-converting enzyme inhibitors (ACEIs), angiotensin receptor blockers (ARBs), β-blockers, diuretics, aldosterone receptor antagonists, digoxin, and anticoagulants, all of which are based on adult HF clinical studies (3). However, none of these pharmacotherapies have demonstrated outcome benefits in children with HF in clinical trials.

Sacubitril/valsartan is a first-in-class angiotensin receptor neprilysin inhibitor that has a novel mechanism of action providing simultaneous inhibition of neprilysin and blockade of the renin-angiotensin aldosterone (RAAS) system. In the PARADIGM-HF trial, sacubitril/valsartan was shown to be superior to enalapril in reducing hospitalizations for worsening HF, cardiovascular mortality, and all-cause mortality in patients with HF with reduced ejection fraction (HFrEF) (4). Symptomatic hypotension was more frequent in patients treated with sacubitril/valsartan than in those treated with enalapril, but this did not affect the clinical benefits of sacubitril/valsartan therapy (5). In the 2021 ESC Guidelines for the diagnosis and treatment of acute and chronic HF, sacubitril/valsartan is recommended as a replacement for an ACEI in patients with HFrEF to reduce the risk of HF hospitalization and death (6).

The PANORAMA-HF study (Prospective trial to assess the Angiotensin Receptor Blocker Neprilysin Inhibitor LCZ696 vs. ACEI for Medical treatment of Pediatric HF) is to determine if sacubitril/valsartan can offer a greater clinical treatment benefit compared to enalapril for pediatric HFrEF patients over 52 weeks treatment duration, as assessed using a global rank endpoint (7). In October 2019, the FDA approved the use of sacubitril/valsartan for use in children older than 1 year of age with symptomatic HFrEF based on a PANORAMA-HF trial which showed reductions in the cardiac biomarker NT-proBNP in pediatric patients.

However, sacubitril/valsartan is not yet approved for pediatric patients in China. By now, only a few centers in China have started to use sacubitril/valsartan to treat children with HF following the PANORAMA-HF protocols. Related clinical results are seldomly reported so far. This study aims to preliminarily evaluate the safety and efficacy of sacubitril/valsartan in children with HF by analyzing the clinical data of patients treated with sacubitril/valsartan at our center since January 2019.

## Methods

Clinical parameters of patients with HF due to CHD or CM prescribed with sacubitril/valsartan at a single center (Shanghai, China) from January 2019 to March 2023 were collected and analyzed retrospectively. The inclusion criteria for the current study consisted of (i) age between 1 month and 18 years, (ii) LVEF <45% by echocardiography, and (iii) sacubitril/valsartan treatment for ≥4 weeks. The exclusion criteria were: (i) age <1 month or ≥18 years, (ii) LVEF ≥45% by echocardiography, or (iii) sacubitril/valsartan treatment for <4 weeks, (iv) loss to follow-up or telephone interview in May 2023.

Using the computerized database of clinical management system, we recorded the date of birth, gender, diagnoses, height, weight, starting time and doses of sacubitril/valsartan medication, and combined medications of all included patients. HF symptoms, NYHA classifications, clinical examinations and laboratory results such as echocardiography parameters and serum NT-proBNP concentrations, as well as occurrences of negative clinical events were collected and analyzed in this study. For the CHD patients who developed HF after cardiac surgeries, we recorded their types of malformations and surgical modalities. Telephone interviews were conducted in May 2023 to acquire latest conditions of enrolled patients. Patients or their families were asked to complete a questionnaire designed to evaluate conditions of HF children, in which their latest height and weight, HF symptoms, exercise ability, re-admissions, survival and transplantation situations, and current medications were put down in record.

The initial dose of sacubitril/valsartan was either 0.2 mg/kg bid or 0.4 mg/kg bid, with a target dose of 2.3 mg/kg bid or 3.1 mg/kg bid, which was in accordance with the PARANOMA-HF design (7). Selection and adjustment of dose for each patient were based on their ages and tolerance. The medication was administered in 100 mg tablets via oral or nasogastric feeding. Patients who had previous ACEI/ARB treatment discontinued their ACEI/ARB at least 36 h before receiving sacubitril/valsartan to prevent adverse reactions. The titration and dose progression were based on overall safety and tolerability. Patients that appeared with drug-related adverse events would experience dose reduction, suspension or discontinuation of medication. Other medications treating HF that were applied to the patients simultaneously were recorded.

Clinical endpoints were used to evaluate the clinical response and efficacy of sacubitril/valsartan in children with HF due to CM and post-operative CHD. The primary endpoint was the LVEF of patients reaching up to 45%, and the secondary endpoints were the decrease in NT-proBNP, changes in NYHA HF classifications, re-admissions due to HF and death or transplantation. Continuous data were presented as mean ± standard deviation if normally distributed or as median (interquartile range) if not normally distributed and categorical variables were presented as percentages. The Student's *t*-test or the Mann–Whitney *U* test was used for comparisons between continuous data, and χ2 test or Wilcoxon test was used for comparisons between categorical data. A two-tailed *P* value < 0.05 was considered to be statistically significant. All the statistical analyses were performed using the IBM SPSS Statistics 25.0 software. *Z*-scores were calculated using the methods illustrated in former reports (8, 9), through the Boston Children's Hospital Heart Center's *Z*-score Calculator which is based on data gathered over the past 12 years on normal children obtained from the downloadable files available from the CDC and WHO. Body weight and height was normalized by age, and echocardiographic data was normalized by body surface area (BSA).

## Results

### Patient characteristics

**Figure 1:**
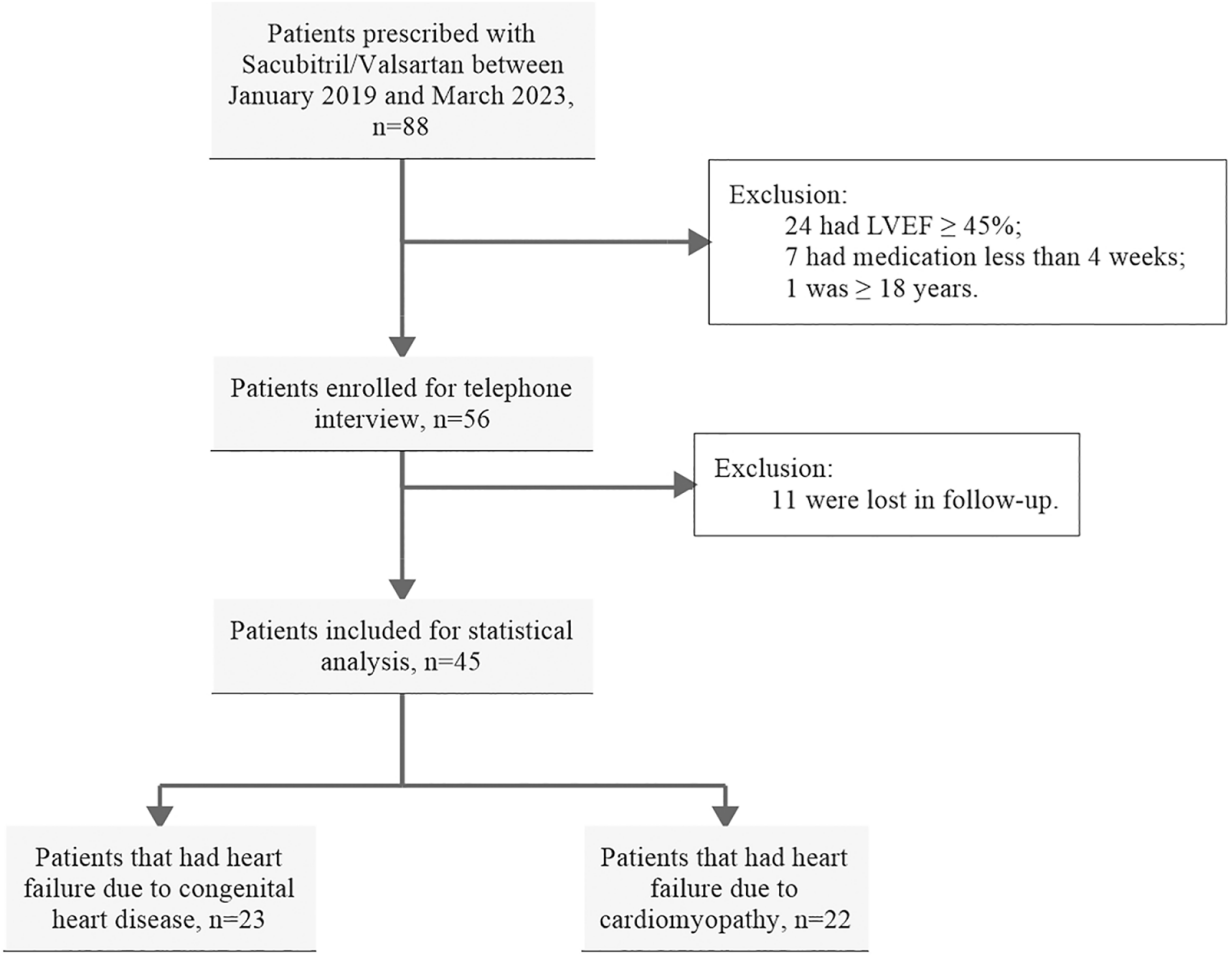
Study enrolment flow chart.

Eighty-eight patients prescribed with sacubitril/valsartan between January 2019 and March 2023 were enrolled in the study. Of these, a total of 43 patients were excluded after applying inclusion and exclusion criteria illustrated before. Therefore, 45 patients were included in the analysis, among which 22 patients had HF due to CM, and 23 patients had HF due to CHD (Figure 1).

### Baseline characteristics

The 45 enrolled patients were 59.1% male and had a median age of 9.52 years (3.40–11.65). Most of the CM patients (18/22) were older than 2 years, while almost a third of the CHD patients (7/23) were younger than 2 years, with the youngest being 3 months old. The different demographic and clinical characteristics of the CM and CHD patient groups were also reflected by differences in body weight, height, BSA, NT-proBNP, NYHA classifications, and echocardiographic parameters as indicated in Table 1, with the comparison between the two etiologies presented by *p*-values. The median follow-up time was 1.23 (0.50–1.87) years, with 1.45 (0.70–1.92) years in the CHD group and 0.74 (0.32–1.39) years in the CM group. The 23 patients who had HF due to CHD developed HF after heart surgeries, during which aortic cross-clamping and cardiopulmonary bypass were involved. 17/23 started taking medication within a month after their surgeries, during the early post-operative period, and 6/23 began the medication in the mid- or late- post-operative period, ranging from 1 month to 1 year after the surgery. Among the CM patients, 19 had dilated cadiomyopathy and 3 had non-compaction cardiomyopathy. The CM group started the medication during the acute onset (progression period) of HF. There were significant differences in the comparison of baselines between the two groups in terms of *z*-score of weight for age (WAZ), left ventricular posterior wall thickness at end-diastole (LVPWd) *Z*-scores, and NT-proBNP levels. In the CM group, the WAZ was smaller (*p* = 0.027), which may be due to the effects of chronic HF on growth; the LVPWd *Z*-scores were lower (*p* = 0.004), indicating that the ventricular walls of CM patients were thinner (because most of them had dilated cardiomyopathies). The NT-proBNP levels in the CHD group were higher (*p* = 0.011), which may be a reflection of the early post-operative state of these patients at the time of this assessment.

**Table 1:** Baseline characteristics of enrolled patients.

Variables	Total (*n* = 45)	CHD (*n* = 23)	CM (*n* = 22)	*P*-value
Male	27 (60.0%)	14 (60.9%)	13 (59.1%)	0.903
Age (years)	7.86 (1.89–11.78)	5.25 (0.94–12.01)	9.52 (3.40–11.65)	0.329
Height (cm)	123.0 (84.0–149.5,)	107 (72–149)	131.5 (96.5–150.75)	0.203
Weight (kg)	20.0 (10.4–34.0)	17.5 (7.5–33)	22.25 (12.78–44.05)	0.134
BSA (m^2^)	0.82 (0.49–1.19)	0.73 (0.39–1.16)	0.89 (0.59–1.36)	0.128
HAZ	−0.22 ± 1.01	−0.42 ± 0.96	−0.02 ± 1.04	0.184
WAZ	−1.18 ± 1.23	−1.57 ± 1.18	−0.77 ± 1.17	0.027
LVEF (%)	30.81 ± 6.77	31.10 ± 7.54	30.51 ± 6.03	0.776
LVdd *Z*-score	5.91 ± 3.81	5.06 ± 3.96	6.76 ± 3.55	0.140
LVds *Z*-score	10.60 ± 4.73	9.72 ± 4.94	11.48 ± 4.45	0.221
LVFS	14.48 ± 3.82	14.24 ± 4.38	14.72 ± 3.24	0.682
LVPWd *Z*-score	0.31 ± 2.52	1.37 ± 2.57	−0.75 ± 2.01	0.004
NT-proBNP (pg/ml)	5,501.5 (2,713.25–12,878.25)	11,254 (3,882–15,911)	3,738 (1,306–6,678.5)	0.011
NYHA Classification				
III	19 (42.2%)	8 (34.8%)	11 (50%)	0.307
IV	26 (57.8%)	15 (65.2%)	11 (50%)	
Follow-up Time	1.23 (0.50–1.87)	1.45 (0.70–1.92)	0.74 (0.32–1.39)	0.054
CHD (post-operative)	23 (51.1%)	23 (100%)		
Volume Overload	* *	14 (60.9%)		
Both Volume and Pressure Overload	* *	6 (26.1%)		
Ischemia	* *	3 (13.0%)		
CM	22 (48.9%)		22 (100%)	
Dilated Cardiomyopathy	* *		19 (86.4%)	
Non-compaction Cardiomyopathy	* *		3 (13.6%)	

CHD, congenital heart disease; CM, cardiomyopathy; BSA, body surface area; HAZ, height for age; WAZ, weight for age; LVEF, left ventricular ejection fraction; LVdd, left ventricular end-diastolic dimension; LVds, left ventricular end-systolic dimension; LVFS, left ventricular fractional shortening; LVPWd, left ventricular posterior wall thickness at end-diastole; NYHA, New York Heart Association.

### Clinical outcomes

During a median follow-up of 1.23 (0.50–1.87) years, the clinical outcomes are evaluated by primary and secondary endpoints (Table 2). 23/45 patients reached the primary endpoint of LVEF ≥45%, with 18/23 from the CHD group, which is notably more than the CM group (*p* < 0.001). The decrease in NT-proBNP was more notable in the CHD group than CM [6,724 (105–12,867) pg/ml vs. 905.5 (51.25–2,676.5) pg/ml, *p* = 0.032]. In terms of NYHA class, 24 (53.3%) improved, 18 (40.0%) unchanged and 3 (6.7%) worsened in total. 7 (31.8%), 12 (54.5%), and 3 (13.6%) of the 22 CM patients were improved, unchanged and worsened respectively, indicating worse outcomes than the CHD group (*p* = 0.007). HF re-admissions were less in the CHD group [2 (8.7%) vs. 11 (50.0%), *p* = 0.002]. 9/45 reached the endpoint of death or transplatation, with 2 from the CHD group and 7 from the CM group (*p* = 0.071).

**Table 2:** Endpoints.

Endpoints	Total (*n* = 45)	CHD (*n* = 23)	CM (*n* = 22)	*P*-value
Primary Endpoint				
LVEF reaching up to 45%	23 (51.1%)	18 (78.3%)	5 (22.7%)	<0.001
Secondary Endpoints				
Decreases of NT-proBNP (pg/ml)	2,648 (105–7,824)	6,724 (105–12,867)	905.5 (51.25–2,676.5)	0.032
Changes in NYHA Classifications				
Improved	24 (53.3%)	17 (73.9%)	7 (31.8%)	0.003
Unchanged	18 (40.0%)	6 (26.1%)	12 (54.5%)	
Worsened	3 (6.7%)	0	3 (13.6%)	
HF Re-admission	13 (28.9%)	2 (8.7%)	11 (50.0%)	0.002
Death or Heart Transplantation	9 (20.0%)	2 (8.7%)	7 (31.8%)	0.071

CHD, congenital heart disease; CM, cardiomyopathy; LVEF, left ventricular ejection fraction; NYHA, New York Heart Association; HF, heart failure.

**Figure 2:**
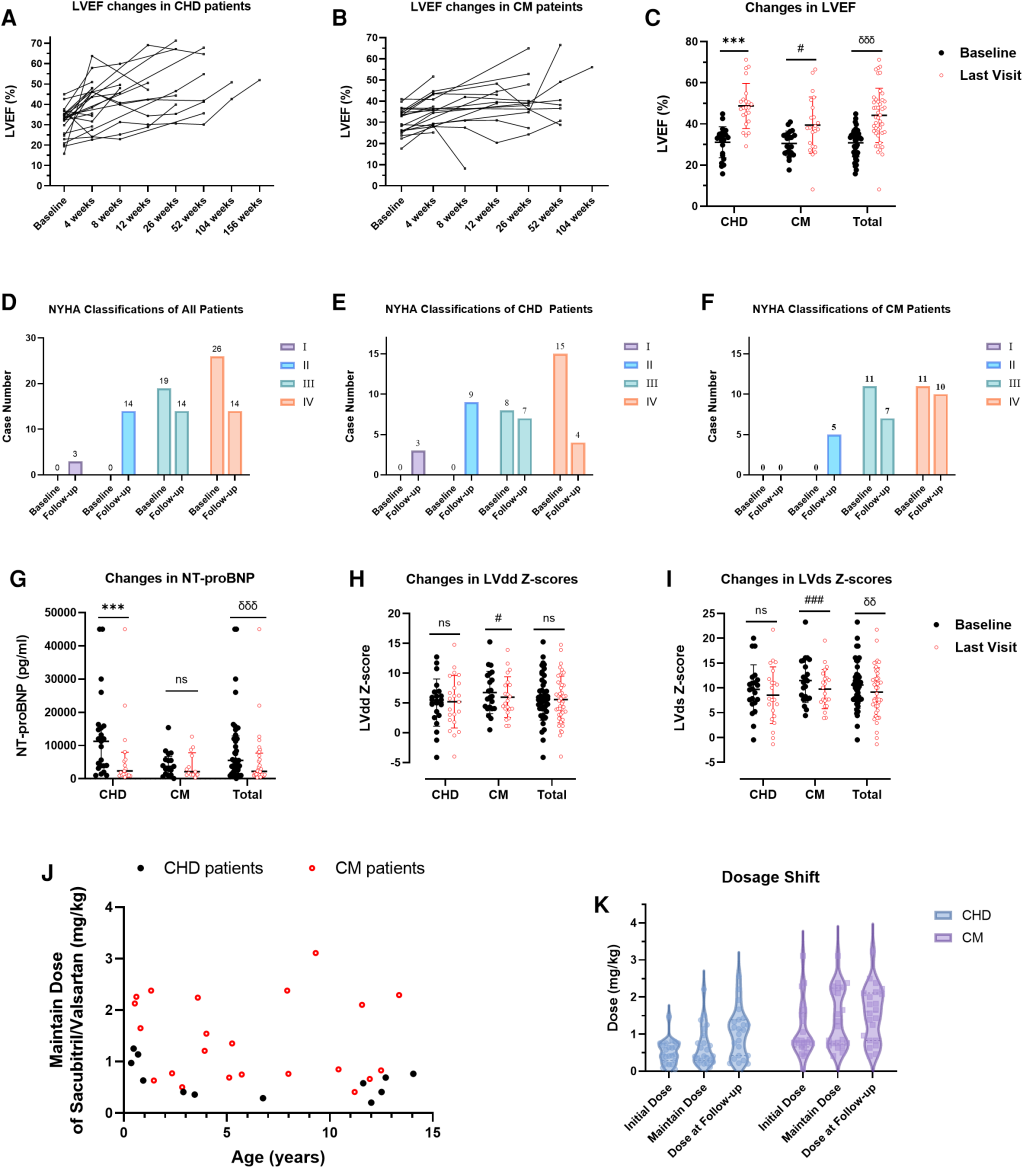
Changes in LVEF vs. time after first administration of sacubitril/valsartan in post-operative CHD patients (**A**) and CM patients (**B**) Changes in LVEF (**C**), NT-proBNP (**G**), LVdd *Z*-scores (**H**), and LVds *Z*-scores (**I**) in all patients and each group respectively. Changes of NYHA classifications in all patients (**D**) and in the two groups respectively (**E,F**). The maintain dose vs. ages of patients in the two groups (**J**). The dosage shift in the two groups (**K**). In (**C,H**) and (**I**) values are means ± SD and comparisons were made through paired sample *t*-tests; in (**G**) values are median (IQR) and comparisons were made through Mann-Whitney *U* tests. Baseline vs. last visit in post-operative congenital heart disease patients: *P < 0.05, **P < 0.01, ***P < 0.001; baseline vs. last visit in cardiomyopathy patients: ^#^*P* < 0.05, ^##^*P* < 0.01, ^###^*P* < 0.001; baseline vs. last visit in all patients: *^δ^P* < 0.05, *^δ^*^δ^*P* < 0.01, *^δ^*^δδ^*P* < 0.001; ns, non-significant.

Figures 2A,B show the changes in LVEF for each patient in the CHD and CM groups, respectively. The mean LVEF increased significantly from 30.81 ± 6.77% to 44.16 ± 13.24% in total (*p* < 0.001), from 31.10 ± 7.54% to 48.91 ± 11.18% in the CHD group (*p* < 0.001), and from 30.51 ± 6.03% to 39.42 ± 13.66% in the CM group (*p* = 0.006), as shown in Figure 2C. Figures 2D–F display the changes in NYHA classifications at follow-up for the total, CHD, and CM patients, respectively. The NT-proBNP levels decreased significantly from 5,501.5 (2,713.25–12,878.25) pg/ml to 2,241.5 (1,164.5–7,726.25) pg/ml in total (*p* < 0.001), and from 11,254 (3,882–15,911) pg/ml to 2,347 (1,030–7,989) pg/ml in the CHD group (*p* = 0.001), as shown in Figure 2G. The decrease in the CM group was not significant, from 3,738 (1,306–6,678.5) pg/ml to 2,136 (1,280.5–7,828) pg/ml (*p* = 0.110). Figures 2H,I show the changes in echocardiographic left ventricular end-diastolic dimensions (LVdd) z- and left ventricular end-systolic dimensions (LVds) *z*-scores, respectively. Supplementary Table S1 provides the clinical data and *p*-values for Figure 2 at baseline and follow-up.

Body weight and height were recorded at follow-up and *z*-scores were calculated to assess the catch-up growth and nutrition state of patients, which could also reflect the severity of HF. During the median follow-up of 1.45 (0.70–1.92) years, we found that the catch-up growth of height and body weight was significant in the CHD group [from −0.42 ± 0.96 to 0.29 ± 1.31 in HAZ (height for age), *p* = 0.025 and from −1.57 ± 1.18 to −0.30 ± 1.46 in WAZ, *p* < 0.001]. CM patients, however, did not show significant improvement during the median follow-up of 0.74 (0.32–1.39) years (Table 3).

**Table 3:** Changes in growth at follow-up.

	Total (*n* = 45)			CHD (*n* = 23)			CM (*n* = 22)		
	Baseline	Follow-up	*P*-value	Baseline	Follow-up	*P*-value	Baseline	Follow-up	*P*-value
Age (years)	7.86 (1.89–11.78)	8.35 (3.69–13.19)		5.25 (0.94–12.01)	7.26 (3.68–12.82)		9.52 (3.40–11.65)	10.68 (3.53–13.25)	
Height (cm)	123.0 (84.0–149.5,)	121.5 (103–158)		107 (72–149)	118 (110–151)		131.5 (96.5–150.75)	124 (99–159.5)	
Weight (kg)	20.0 (10.4–34.0)	22.25 (15.88–45)		17.5 (7.5–33)	22.5 (15.5–41)		22.25 (12.78–44.05)	20 (15.75–48)	
BSA (m^2^)	0.82 (0.49–1.19)	0.82 (0.67–1.36)		0.73 (0.39–1.16)	0.83 (0.68–1.33)		0.89 (0.59–1.36)	0.77 (0.63–1.41)	
HAZ	−0.22 ± 1.01	0.09 ± 1.56	0.190	−0.42 ± 0.96	0.29 ± 1.31	0.025	−0.02 ± 1.04	−0.17 ± 1.83	0.774
WAZ	−1.18 ± 1.23	−0.62 ± 1.57	0.036	−1.57 ± 1.18	−0.30 ± 1.46	<0.001	−0.77 ± 1.17	−1.02 ± 1.66	0.087

CHD, congenital heart disease; CM, cardiomyopathy; BSA, body surface area; HAZ, height for age; WZA, weight for age.

### Dosage shift

The maintain dose of sacubitril/valsartan each patient had during hospitalization and its relationship with the ages of patients is shown in Figure 2J. Upon the latest follow-up, 34 patients were still having daily dose of sacubitril/valsartan, and 11 patients discontinued or transferred to other medications because of intolerance of the medicine or remission of HF. The mean dose reached 1.29 ± 0.74 mg/kg at the last visit, with the dose titration shown in Figure 2K. The dose of administration in the two groups differed significantly, as is indicated in Supplementary Table S2. Other medications at discharge and follow-up are depicted in Table 4.

**Table 4:** Medications at discharge and follow-up.

Medications	Discharge (*n* = 45)	Follow-up (*n* = 45)
Sacubitril/valsartan	45 (100.0%)	32 (71.1%)
Digoxin	21 (46.7%)	16 (35.6%)
Diuretic	38 (84.4%)	26 (57.8%)
Anticoagulation/Antiplatelet	30 (66.7%)	18 (40.0%)
β-blockade	23 (51.1%)	16 (35.6%)
ACEI/ARB	0	3 (6.7%)
Ivabradine	8 (17.8%)	5 (11.1%)
Amiodarone	1 (2.2%)	2 (4.4%)
Glucocorticoid	7 (15.6%)	2 (4.4%)
SGLT2 Inhibitors	4 (8.9%)	1 (2.2%)

ACEI, angiotensin-converting enzyme inhibitor; ARB, angiotensin receptor blocker; SGLT2, sodium glucose cotransporter 2.

### Safety

Safety of the medication is evaluated by the records of adverse events experienced. Patients that had HF due to CM got comparatively higher dose of sacubitril/valsartan than CHD patients, and went through fewer drug-related side effects. Of the17 early post-operative CHD patients who received intravenous diuretics along with the medication, 7 experienced dose reduction or suspension of medication due to hypotension, while CM and mid- or late- post-operative CHD patients who received oral diuretics didn't experience any. Seventeen early post-operative CHD patients started medication within a month after surgeries while maintaining a negative fluid balance state. Among them, 7 patients reported hypotension. However, for CM patients and mid- to late- post-operative CHD patients, diuretics were orally administered and the patients were in a positive fluid balance state, which might be one of the factors contributing to the absence of reports of hypotension among these patients. At their last follow-up, the CHD group and CM group had serum urea nitrogen levels of 7.05 (4.675–10.8) mmol/L and 5.0 (3.9–6.5) mmol/L, respectively (*p* = 0.046), and serum creatinine levels of 29 (29–37.5) μmol/L and 34 (29–46.5) μmol/L, respectively (*p* = 0.098), both of each group within the normal reference range. No other adverse effects such as hyperkalemia, allergy, angioedema, coughs, renal insufficiency or hepatic impair were reported.

## Discussion

In our follow-up of HF children using sacubitril/valsartan, the response of the CHD group seemed to be better (possibly related to surgical benefits). Post-operative CHD patients showed relatively good and even curable recovery, while the course of CM patients exhibited delayed deterioration, with the majority of cases remaining unchanged and even some showing improvement in terms of NYHA classifications. The post-operative CHD patients achieved lower doses and experienced more drug-related adverse events, mainly dose reduction and medication interruption arisen from hypotension. Sacubitril/valsartan was preliminarily shown to improve the quality of life in children with HF, although the mid- and long-term effects remained to be seen.

Although sacubitril/valsartan is widely used in adult HF, it is a relatively new drug in the medical treatment of pediatric HF. The current study focused on the two main etiologies of pediatric HF, CHD and CM, and investigated the difference in the effect of sacubitril/valsartan on HF resulting from the two causes.

The mean dose of sacubitril/valsartan in the last visit was 1.29 ± 0.74 mg/kg, which still did not reach the target dose suggested by the PANORAMA-HF protocols. The CM group had a higher mean dose of 1.56 ± 0.75 mg/kg than the CHD group with 1.03 ± 0.66 mg/kg (*p* = 0.016), as well as fewer adverse effects. However, the outcomes were favorable in terms of NYHA classifications in post-operative CHD patients. This suggested that children with HF who received sacubitril/valsartan might actually require a lower dose and a slower dose up-titration in the real-world setting, especially for post-operative CHD patients who were younger and more susceptible to hypotension, which also appeared to be the most common side effect in adults (4). Out of the 17 patients with CHD who were administered the medication in the early post-operative phase, the primary cause was acute HF. Due to the necessity of strict fluid management following cardiac surgeries, there was a higher likelihood of hypotension occurring, which may result in the reduction or suspension of medication. It has been reported that if sacubitril/valsartan was titrated gradually, the target dose of 200 mg twice daily could be achieved and maintained in a high percentage of adult patients (10). Therefore, we can postulate that establishing scheduled drug-escalation plans might be helpful in achieving the target dose of sacubitril/valsartan in children with HF. Children who develop HF in the early post-operative period after cardiac surgery may begin with a very low dose of medication, which can be gradually increased over a certain titration period while closely monitoring their blood pressure.

In recent years, reversing ventricular remodeling has been a key goal for treating HFrEF patients. Ventricular remodeling refers to changes in the ventricular structure. Echocardiography shows atrial and ventricular lengthening, increases in volume and mass, and deterioration in systolic and diastolic function in adverse remodeling (11). Previous studies used different echocardiographic measures to assess left ventricular reverse remodeling, such as increased LVEF and smaller left ventricular size (12). The current study utilized echocardiographic measurements of LVEF, LVdd *z*-score and LVds *z*-score to evaluate the cardiac function and ventricular size, providing insight into the degree of ventricular remodeling in HF children.

Current evidence shows that sacubitril/valsartan reverse and delay ventricular remodeling and improve patients' quality of life in patients with HF after myocardial infarction (13). In the past few years, several studies reported sacubitril/valsartan could ameliorate HF by reversing ventricular remodeling in animal models (14–17), from which we can postulate that sacubitril/valsartan might play a role in attenuating the process of ventricular remodeling in HF children. Moreover, it is reported that sacubitril/valsartan might attenuate right ventricular modeling and benefit patients with right heart overload or dysfunction (18–21), which is also proved in animal models in the latest researches (22).

In the current study, both subgroups achieved significant improvement in LVEF. In the CHD group, LVEF improved from 31.10 ± 7.54% to 48.91 ± 11.18% (*p* < 0.001), while in the CM group, LVEF improved from 30.51 ± 6.03% to 39.42 ± 13.66% (*p* = 0.006). The post-operative CHD patients with HF had significantly better performance in achieving the primary endpoint of 45% LVEF compared with children with CM (*p* < 0.001). On the other hand, CM patients demonstrated significant improvement in the BSA normalized ventricular dimensions (*p* = 0.037 for LVdd *z*-score and *p* < 0.001 for LVds *z*-score) while the CHD patients didn’t, which indicated the slowdown of the progression of disease in the CM group.

NT-proBNP also helps in assessing cardiac remodeling in HFrEF patients (11).In the current study, the median serum NT-proBNP levels decreased from 5,501.5 (2,713.25–12,878.25) pg/ml to 2,241.5 (1,164.5–7,726.25) pg/ml (*p* < 0.001). In the CHD group, the NT-proBNP levels decreased significantly from 11,254 (3,882–15,911) pg/ml to 2,347 (1,030–7,989) pg/ml (*p* = 0.001). The CM group decreased from 3,738 (1,306–6,678.5) pg/ml to 2,136 (1,280.5–7,828) pg/ml, although not statistically significant (*p* = 0.110). The decrease of NT-proBNP in the post-operative CHD group was more remarkable than that in the CM group (*p* = 0.043). Given the significant difference in baseline NT-proBNP between the two groups (*p* = 0.011) and the fact that the CHD group had a median baseline as high as 11,254 pg/ml due to acute post-operative response, the difference in NT-proBNP improvement between the two groups is understandable. Although the improvement of NT-proBNP was not statistically significant after the prescription of sacubitril/valsartan in CM patients, the median level appeared to have decreased from 3,738 pg/ml to 2,136 pg/ml. In a former study, NT-proBNP demonstrated as a strong independent predictor for adverse outcome in children with dilated cardiomyopathy (23). What's more, the reduction in NT-proBNP along with an improvement in echocardiography can better indicate the reverse in left ventricular remodeling and is associated with improved clinical outcomes (11).

The degree of heart failure in the children was also indicated by their nutritional status and catch-up growth during follow-up. Both HAZ and WAZ of children in the CHD group were significantly improved at the last visit compared to baseline (*p* = 0.025 in HAZ and *p* < 0.001 in WAZ). The CM group on the other hand, didn't gain improvement in HAZ nor WAZ, which implied poorer catch-up growth and worse HF recovery in this group. Although not statistically significant (*p* = 0.054), there was a notable difference in follow-up time between the two groups. The CHD group had a follow-up time of 1.45 (0.70–1.92) years, while the CM group had a follow-up time of 0.74 (0.32–1.39) years (Table 1), which may be one contributing factor to the better catch-up growth observed in the CHD group.

In the US, nearly 40% of children with symptomatic CM either undergo heart transplantation or die within two years (24). In the current study, 9/45 patients ended in death or heart transplantation by the last visit, 7 of which being CM patients. The CM patients started medication at an advanced stage of disease during the acute onset of HF, with 8/22 (34.8%) at class III and 15/22 (65.2%) at class IV of NYHA classification. During a median follow-up of 0.74 (0.32–1.39) years, 7/22 (31.8%) of these patients reached the endpoint of death or heart transplantation. The NYHA classification improved in general and in the post-operative CHD group (both *p* < 0.001), while the CM group did not show significant improvement in the NYHA class, with 12/22 unchanged and 3/22 worsened. In the CHD group, there was a difference in changes between the early and mid- to late- post-operative groups in terms of NYHA classification. The median improvement in NYHA for early post-operative patients was 1 (0.5–2), while the median improvement for mid- to late- post-operative patients was 0.5 (0–1) (*p* = 0.045). The early post-operative patients had significantly greater improvement of NYHA class than the CM group (*p* = 0.001), while the mid- to late- post-operative patients didn't (*p* = 0.405). This implies that early post-operative CHD patients might achieve the cure of acute HF, while chronic HF in mid- to late- post-operative patients tends to have a worse prognosis, more closely resembling that of CM patients.

Moreover, 11 of the 22 CM patients were re-admitted due to HF, which was significantly higher than that of the post-operative CHD patients (*p* = 0.002). These all indicate worse clinical outcomes as well as quality of life in the CM group than the CHD group. This might be attributed to the fact that HF in post-operative CHD patients was mostly acute, transient and thus partially reversible. On the other hand, the CM patients developed HF as a result of myocardial abnormalities, and medical therapies had limited effects, especially for end-stage dilated cardiomyopathies, leaving heart transplantation as the main definitive treatment (25). However, a recent report showed that the mortality of pediatric dilated cardiomyopathies was significantly lower between 2000 and 2010 than between 1990 and 2000 (26), possibly as a result of nontransplantation therapies improving survival and overall advances in treatment for children with acute and chronic HF, highlighting the importance of searching for new effective drugs to treat pediatric HF. The current study proposes that sacubitril/valsartan may be a promising potential medication for treating HF in children.

## Limitations

Several limitations should be addressed in the present study. First, the design was a single-center retrospective observational study and involved a relatively small number of patients without a control group that received a different medication for comparison. The study also divided the patients into subgroups based on the direct cause of their HF, but some patients might have had more than one cause, such as CHD combined with CM. Moreover, most of the patients were hospitalized for HF when they started sacubitril/valsartan and they might have had fluid retention which could affect the accuracy of their BSA and *z*-scores of their body weight. Despite these limitations, the study used reliable echocardiographic and laboratory parameters to measure the effects of sacubitril/valsartan on ventricular function and HF severity in children. Therefore, randomized controlled trials are needed to evaluate the mid- and long-term effects of sacubitril/valsartan on children with HF and more basic research is required to elucidate the mechanisms of how sacubitril/valsartan affects HF in children.

## Conclusions

Overall, in this follow-up study of HF children using sacubitril/valsartan, post-operative CHD patients showed significant improvement in echocardiographic LVEF, NT-proBNP levels, NYHA classes and catch-up growth, while CM-induced HF patients demonstrated significant improvement in LVEF and BSA normalized LVdd and LVds. In patients that develop HF in the early post-operative period following CHD surgeries, sacubitril/valsartan may be considered as a treatment option which may perform an add-on therapeutic effect, with administration beginning at a very low dose and gradually increasing to avoid hypotension. These patients have the potential to achieve a clinical cure for surgery-related HF. However, medication for HF that occurs in the mid- to late- post-operative period in CHD patients is similar to that of the CM patients, which may only delay the deterioration of cardiac function.

## Data Availability

The original contributions presented in the study are included in the article/**Supplementary Material**, further inquiries can be directed to the corresponding author/s.
