# Supplementary material for: Does sacubitril/valsartan work in children with heart failure?—a pilot study
Source: Front Cardiovasc Med. 2023 Nov 29;10:1274990. doi: 10.3389/fcvm.2023.1274990 (PMC10716201; doi:10.3389/fcvm.2023.1274990)
Supplement: Supplementary file 1 [file Table1.pdf]

Supplement Materials

Table S1. Changes of clinical parameters between baseline and last visit

	Total (n=45)			CHD (n=23)			CM (n=22)		
	**Baseline**	**Last Visit**	**P-value**	**Baseline**	**Last Visit**	**P-value**	**Baseline**	**Last Visit**	**P-value**
**LVEF (%)**	30.81±6.77	44.16±13.24	<0.001	31.10±7.54	48.91±11.18	<0.001	30.51±6.03	39.42±13.66	0.006
**LVdd Z-score**	5.91±3.81	5.58±3.92	0.345	5.06±3.96	5.20±4.41	0.810	6.76±3.55	5.96±3.42	0.037
**LVds Z-score**	10.60±4.73	9.15±4.89	0.003	9.72±4.94	8.54±5.71	0.177	11.48±4.45	9.77±3.94	<0.001
**LVFS (%)**	14.48±3.82	22.39±7.72	<0.001	14.24±4.38	24.87±6.91	<0.001	14.72±3.24	19.91±1.67	0.005
**LVPWd Z-score**	0.31±2.52	0.41±2.73	0.812	1.37±2.57	0.98±2.53	0.380	-0.75±2.01	-0.18±2.86	0.399
**NT-proBNP (ng/ml)**	5501.5 (2713.25~12878.25)	2241.5 (1164.5~7726.25)	<0.001	11254 (3882~15911)	2347 (1030~7989)	0.001	3738 (1306~6678.5)	2136 (1280.5~7828)	0.110
**NYHA Classification**			<0.001			<0.001			0.130
I	0	3 (6.7%)		0	3 (13.0%)		0	0	
II	0	14 (31.1%)		0	9 (39.1%)		0	5 (22.7%)	
III	19 (42.2%)	14 (31.1%)		8 (34.8%)	7 (30.4%)		11 (50%)	7 (31.8%)	
IV	26 (57.8%)	14 (31.1%)		15 (65.2%)	4 (17.4%)		11 (50%)	10 (45.5%)	

CHD, congenital heart disease; CM, cardiomyopathy; LVEF, left ventricular ejection fraction; LVdd, left ventricular end-diastolic dimension; LVds, left ventricular end-systolic dimension; LVFS, left ventricular fractional shortening; LVPWd, left ventricular posterior wall thickness at end-diastole; NYHA, New York Heart Association.

Table S2. Dosage of sacubitril/valsartan during follow-up

**Dose of Sacubitril/Valsartan**	**Total (n=45)**	**CHD (n=23)**	**CM (n=22)**	**P-value**
**Initial Dose (mg/kg)**	0.69 (0.41~0.84)	0.46 (0.29~0.76)	0.80 (0.65~1.55)	< 0.001
**Maintain Dose (mg/kg)**	0.76 (0.41~1.53)	0.41 (0.29~0.97)	1.28 (0.74~2.25)	< 0.001
**Dose at Follow-up (mg/kg)**	1.29±0.74	1.03±0.66	1.56±0.75	0.016
